# To First Year's Men

**Published:** 1892-09-24

**Authors:** 


### To First Year's Men.

When a medical student enters on his work which is to lead him to his *summum bonum*—a double qualification to practice—he is always impressed with the stupendous amount of knowledge he is expected to acquire even in the preliminary stages of his education. The fact is, the lines of thought are so altogether fresh to him that his studies appear to be much more difficult than is really the case, and just at first his inability to acquire and retain the numerous facts and theories of anatomy and physiology and other sciences with which the older students seem so familiar is disappointing and discouraging. Now, many a student is made or marred at the outset for these very reasons. There are very few indeed who do not enter upon their student life full of hope for the future, and with excellent intentions to work steadily, but the new life presents many temptations to put off the steady work till the morrow, and this too frequently means till the following session. Consequently two winters' work must be stuffed into one. Naturally, this is beyond the capability of the average mind, and the "exam." must be worked up to. The student can only afford to learn "what is asked at the college," and his one desire is to scrape through. For that man the chances are that the whole five years set apart for studying medicine are spoilt, even though he may never fail at a single examination.

Students, your life's work depends upon the acquirement of a sound practical and theoretical knowledge of the medical art, and if you choose to be haunted by that eternal bogey "the exam.," you have yourselves to thank for spoiling what should be the five happiest years of your lives. The whole of that period has been carefully mapped out and divided up for you, and any student of ordinary intelligence can pass all the ordinary examinations without fear of failure, if he will only make up his mind to act like a man of sense. Do neither more nor less than each day's work, but do it faithfully; and when, in good time, the examination day arrives, you will enter upon it with confidence, born of a sense of thorough knowledge and patient work. Your examiners are not fools: they have all been through the mill themselves: and if you know your work they will certainly find it out.

Our remarks are not addressed to the few essentially idle and vicious that are found amongst medical students, as well as all other large bodies of young men, but to those who, with every intention to do well, miss the golden chances of life because they forget that deeds, not words, acts, not intent, are essential to success. We have known students who even deny themselves all legitimate pleasure and relaxation, who surround themselves with books and note books, and are always going to begin to work tremendously, are always planning the grand campaign upon which they never enter.

On the other hand, we cannot too strongly urge less work and more play on those foolish young men who forget the duty of maintaining their health and bodily vigour. The work of a medical practitioner is peculiarly arduous and trying, and though the weakly by nature are constantly falling a ready prey to the insidious dangers of infectious disease, they far more frequently succumb from sheer wear and tear. After all it is success in life, not merely success in passing a few examinations that we should aim at, and without health it is impossible to attain the fullest measure of success in the medical profession.

Work steadily, but, oh! play steadily, day by day, if you would enjoy success in your student days, and obtain all that is best worth having in after life. Join the school football club, the cricket club, or play fives, tennis, go to a gymnasium, or, at any rate, have some definite form of healthy exercise which you can take up with energy and enthusiasm.

And in your work don't be fool enough to imagine that you know what to do with your time better than those who have planned your course of study. Don't cut lectures because you "can get it all out of the books." Hearing lectures on a subject is one thing, reading the matter up for yourself another, and seeing, observing, and doing in the dissecting-room and the hospital wards another. You require all three methods of studying your profession, and you will certainly fail to get the best training as a medical man if you are so wise in your own conceit as to neglect any one of the methods of acquiring your art that generations of experience have led the great body of medical practitioners to consider essential to that end.

